# Supplementary material for: Disrupted Mitochondrial Network Drives Deficits of Learning and Memory in a Mouse Model of FOXP1 Haploinsufficiency
Source: Genes (Basel). 2022 Jan 11;13(1):127. doi: 10.3390/genes13010127 (PMC8775322; doi:10.3390/genes13010127)
Supplement: Supplementary file 1 [file genes-13-00127-s001.zip › genes-1532028-supplementary.pdf]

Table S1: Primer list

Genes	Sequence
Foxp1 430bp 1	5'-CCTCTGGCGATGAACCTAGTGGT-3'
Foxp1 430bp 2	5'-AGCCACACTTTCTCTCAGGATGT-3'
Foxp1 280bp 1	5'-AGCGCATGCTCCAGACTGCCTTG-3'
Foxp1	For 5'-AGAGCGCCTGCAAGCCATGA-3'
	Rev 5'-GGCGGTGGGGGTTGTTGGAG-3'
Foxo1	For 5'-AAGAGCGTGCCCTACTTCAA-3'
	Rev 5'-CTCCCTCTGGATTGAGCATC-3'
Hprt1	For 5'-TCCTCCTCAGACCGCTTTT-3'
	Rev 5'-CCTGGTTCATCATCGCTAATC-3'
Sdha1	For 5'-CATGCCAGGGAAGATTACAAA-3'
	Rev 5'-GTTCCCCAAACGGCTTCT-3'
Pgc-1α	For 5'-GGACGGAAGCAATTTTTCAA-3'
	Rev 5'-TTACCTGCGCAAGCTTCTCT-3'
Tfam	For 5'-TCTGTCTCCTGAGGAAAAGCAG-3'
	Rev 5'-ACTTCGTCCAACTTCAGCCA-3'
D-loop	For 5'-GACCAACATAACTGTGGTGTCA-3'
	Rev 5'-ATTCTTCTCCGTAGGTGCGTCTAG-3'
16srRNA	For 5'-CCGCAAGGGAAAGATGAAAGAC-3'
	Rev 5'-TCGTTTGGTTTCGGGGTTTC-3'
Nd1	For 5'-CTAGCAGAAACAAACCGGGC-3'
	Rev 5'-CCGGCTGCGTATTCTACGTT-3'
B2m	For 5'-TGTCAGATATGTCCTTCAGCAAGG-3'
	Rev 5'-TGCTTAACTCTGCAGGCGTATG-3'
Hk2	For 5'-GCCAGCCTCTCCTGATTTTAGTGT-3'
	Rev 5'-GGGAACACAAAAGACCTCTTCTGG-3'

Table S2: Antibody list

Primary Antibody	Supplier	Application& dilution	Catalog #	RRID
Anti-Bcl-2 antibody	Abcam	WB 1:1000	ab692	AB_305670
Anti-FOXP1 antibody (rabbit)	Abcam	WB 1:1000	ab16645	AB_732428
Anti-PGC-1 Alpha antibody	Abcam	WB 1:1000	ab54481	AB_881987
Anti-Mitofusin 1 antibody	Abcam	WB 1:1000	ab104274	AB_10712138
Anti-TFAM antibody	Abcam	WB 1:1000	ab131607	AB_11154693
Anti-TTC11/FIS1 antibody	Abcam	WB 1:1000	ab71498	AB_1271360
Anti-GAPDH rabbit antibody	Abcam	WB 1:5000	ab9485	AB_307275
Anti-GAPDH mouse antibody	Abcam	WB 1:5000	ab8245	AB_2107448
COX IV Antibody	Cell Signaling Technology	WB 1:1000	4844S	AB_2085427
Foxo1 Rabbit mAb	Cell Signaling Technology	WB 1:1000	2880S	AB_2106495
LC3A/B (D3U4C) Rabbit mAb	Cell Signaling Technology	WB 1:1000	12741S	AB_2617131
Purified Mouse Anti-OPA1	BD Biosciences	WB 1:1000	612606	AB_399888
Purified Mouse Anti-DLP1	BD Biosciences	WB 1:1000	611112	AB_398423
Parkin (Prk8) Mouse mAb	Cell Signaling Technology	WB 1:1000	4211S	AB_2159920
PINK1 rabbit polyclonal antibody	Proteintech	WB 1:1000	23274-1-AP	AB_2879244
Purified Mouse Anti-Cytochrome C	BD Biosciences	WB 1:1000	556433	AB_396417
Recombinant Anti-Bax antibody [E63]	Abcam	WB 1:1000	ab32503	AB_725632
SOD2 antibody	GeneTex	WB 1:1000	GTX116093	AB_10624558

Secondary Antibody	Supplier	Application& dilution	Catalog #	RRID
IRDye 800CW Donkey anti-Rabbit	LI-COR Biosciences	WB 1:10000	926-32213	AB_621848
IRDye 680CW Donkey anti-Mouse	LI-COR Biosciences	WB 1:10000	926-68072	AB_10953628
